# Interaction of sleep quality and anxiety on quality of life in individuals with type 2 diabetes mellitus

**DOI:** 10.1186/s12955-020-01406-z

**Published:** 2020-05-24

**Authors:** Dong Dong, Peian Lou, Jian Wang, Pan Zhang, Jianquan Sun, Guiqiu Chang, Chunrong Xu

**Affiliations:** 1 Department of Endocrinology, Xuzhou Third People’s Hospital, 131 Huancheng Road, Xuzhou, China; 2 Department of Control and Prevention of Chronic non-communicable diseases of Xuzhou Center for Disease Control and Prevention, The School of Public Health, Xuzhou Medical University, 142 West Erhuan Road, Xuzhou, 221006 China

**Keywords:** Type 2 diabetes mellitus, Quality of life, Interaction

## Abstract

**Purpose:**

Sleep disturbances and anxious symptoms are very common in people with type 2 diabetes mellitus(T2DM). This study aimed to assess the interactive effects of poor sleep quality and anxious symptoms on the quality of life of people with T2DM.

**Methods:**

Nine hundred and forty-four participants with T2DM were enrolled in a cross-sectional study. Demographic and physiological characteristics were recorded. Each participant completed a Chinese version of the Pittsburgh Sleep Quality Index, the Patient Health Questionnaire-9 and General Anxiety Disorder questionnaire, and the Diabetes Specificity Quality of Life scale. The products of poor sleep quality and anxiety were added to a logistic regression model to evaluate the multiplicative interactions, expressed as the relative excess risk of interaction, the attributable proportion of interaction, and the synergy index.

**Results:**

Poor sleep quality and anxiety symptoms were associated with reduced quality of life. There was a significant interaction between poor sleep quality and anxiety symptoms; this combined effect significantly reduced quality of life scores by 6.09-fold. The relative excess risk of interactions was 1.36.

**Conclusions:**

The combined effect of poor sleep quality and anxiety symptoms reduces quality of life in people with T2DM.

**Trial registration:**

ChiCTR-IOP-16008045. Registered 3 March 2016.

A clinical study to investigate gum infection in patients undergoing kidney dialysis.

## Introduction

The prevalence of diabetes has been increasing globally, with an estimated number of 592 million diabetes cases by 2035 [1]. In China, more than 100 million people (approximately 1 in 10) have diabetes [2]. Because of unstable glycemic control, diabetes can affect any organ in the body, leading to serious complications over time [3]. Diabetes and its complications result in significant economic burden for individuals, families, and health care systems. The rate of glycemic control in T2DM is only 17.4% in China [4]. To maintain control of blood glucose and reduce complications, people with diabetes require lifelong dietary restrictions, physical exercise, medication compliance, and blood glucose monitoring [5]. During this long-term, self-management process, many individuals experience emotional burdens and poor sleep quality in response to these prolonged requirements, including worry about complications, fear of hypoglycemia, feelings of guilt about uncontrolled blood glucose, and depressed mood [6].

Anxiety is one of the most common comorbidities of people with T2DM [7] and its prevalence ranges from 22.4 to 75% [8]. In people with T2DM, comorbid anxiety disorders are associated with an increase in complications [8], poorer blood glucose levels [8], poor adherence to treatment [9], reduced quality of life [7]. Sleep disturbances are very common symptoms in people with T2DM, who show a 30 to 50% prevalence of poor sleep quality [10]. Sleep disturbance in people with T2DM can result in impaired glucose regulation and quality of life [11]. Even after controlling for age, years since diabetes diagnosis, number of comorbidities, number of diabetic complications, insulin use and depressive symptoms, Poor sleep quality in people with T2DM still significantly reduce diabetes-related quality of life [12].

Although anxiety and poor sleep quality have been shown to be individually correlated with quality of life in people with T2DM, no studies have addressed the joint effects of these factors on T2DM. Hence, the aim of this study was to examine the joint effects of poor sleep quality and anxiety symptoms on quality of life of people with T2DM in primary care in China.

## Materials and methods

### Study design

Xuzhou City locates in northern Jiangsu Province, eastern part of China, and is moderately well-developed with a population of 10,000,000 across all area. More than 24 ten thousand persons with T2DM have registered in 3980 communities of Xuzhou City, average number of 60 patients in each community. The methods that enrolled participants in the study had been described previously [4]. The prevalence of anxiety is higher than that of poor sleep quality in people with T2DM in China [4, 13]. Considering 30% prevalence of poor sleep quality in patients with T2DM [4] with a 90% power and α = 0.05, and allowing for a refusal of 10%, at least 900 people must be selected. The study was conducted in September 2017.

### Inclusion and exclusion criteria

Participants had to meet the recommended criteria of the Chinese Type 2 Diabetes Prevention and Control 2010 Guidelines [3]. The following exclusion criteria were used: (1) participant was diagnosed with Type 2 diabetes mellitus by physicians at least 6 months; (2) Type 1 diabetes mellitus; (3) painful diabetic sensory neuropathy; (4) diagnosed sleep disorders prior to diabetes; (5) mental illness or use of any kind of psychotropic medication; (6) working night shifts in the last 3 months or travelling across time zones in the last month; (7) other endocrine disorders, such as thyroid disease or chronic use of glucocorticoids; (8) age < 18 years; (9) pregnancy or lactation,(10) medically unstable condition;(11) harmful or hazardous alcohol user; (12) Receiving sleep treatment; (13) Another sleep disorders such as obstructive sleep apnea.

A total of 1056 eligible T2DM patients aged 27–97 years old were invited into the study. Among them 23 patients dropped out due to working, 27 patients refused to take part in the study, and nine patients failed to complete the questionnaire. Finally, 997 individuals with T2DM from 44 communities who met the criteria were enrolled in the study. The response rate was 94.4%.

Written informed consent was obtained from all participants. The study protocol was approved by Xuzhou Center for Disease Control and Prevention. The procedures followed were in accordance with the standards of the ethics committee of Xuzhou Center for Disease Control and Prevention and with the Declaration of Helsinki (1975, revised 2000). The trial was prospectively registered with the Chinese Clinical Trials Registry (reference: ChiCTR-IOP-16008045).

### Sleep quality

The Pittsburgh Sleep Quality Index (PSQI) is a 19-item, self-report questionnaire that measures sleep quality and degree of sleep difficulties over the past month [14]. It contains seven component scores: subjective sleep quality, sleep latency, sleep duration, habitual sleep efficiency, sleep disturbances, use of sleep medications, and daytime dysfunction. These are summed to yield a total PSQI score with a range of 0–21; higher scores indicate poorer sleep quality. The Chinese version of the PSQI has a diagnostic sensitivity of 98.3% and specificity of 90.2% in distinguishing people with and without sleep quality problems [15]. In our study, a PSQI score of < 7 and ≥ 7 was defined as “good sleep quality” and “poor sleep quality,” respectively.

### Anxiety and depression

Depression symptoms were assessed with the Patient Health Questionnaire-9 (PHQ-9), a nine item scale with scores ranging from 0 to 27; scores of 10 or more are indicative of depressive symptoms [16]. The Chinese version of PHQ-9 had been well validated in many studies [17–19] and demonstrated a good internal consistency in those study, with a Cronbach’s α coefficient of 0.81. Anxiety Symptoms were measured with the validated seven-item General Anxiety Disorder questionnaire (GAD-7; total score range 0–21) [20], and participants were defined as having the anxious symptoms if they scored ≥10.The Chinese version of GAD-7 has been widely used and well validated in many studies [21, 22] and demonstrated good internal consistency in those study, with a Cronbach’s α coefficient of 0.88.

### Quality of life

The Diabetes Specificity Quality of Life Scale (DSQL) was used to assess quality of life. This is a validated questionnaire developed by Chen et al. [23] to assess people with Type 2 diabetes mellitus in China. The scale contains 24 items, which generate four domain scores reflecting physiological, psychological, social, and therapy-related problems with quality of life. The sum of the four domain scores produces a global quality of life score with a range of 24–120 points; higher scores indicate poorer quality of life. A DSQL score < 40 has a diagnostic sensitivity of 94.5% and specificity of 91.0% in differentiating good from poor quality of life [23].

### Other variables

We designed an additional questionnaire to record age, sex, level of education, marriage status, physical activities, net household income, cigarette smoking, alcohol consumption, years since diabetes diagnosis, number of comorbidities, number of diabetic complications, and insulin use. The number of diabetic complications was determined by participants’ reports of diagnosed coronary artery disease, peripheral vascular disease, stroke, nephropathy, retinopathy, or neuropathy. All fasting venous blood samples were drawn between 8:00 AM and 9:00 AM. We used the level of HbA1c as the index for glycemic control. HbA1c level was assayed using high-performance liquid chromatography (BIO-RAD Diagnostic Group, CA, USA). A level of HbA1c of 53 mmol/mol (< 7.0%) was defined as good glycemic control based on the Chinese Type 2 Diabetes Prevention and Control 2010 Guidelines and a level of HbA1c of ≥7.0%(53 mmol/mol) was considered poor glycemic control [3]. Body weight and height were measured to the nearest 0.1 kg and 0.1 cm in light indoor clothing, respectively. Body mass index (BMI) was calculated as weight (kg) divided by the square of the height (meters), and categorized as underweight (< 18.5 kg/m^2^), normal weight (18.5–23.9 kg/m^2^), and overweight/obese (≥24.0 kg/m^2^) [24]. Alcohol user was assessed by the Alcohol Use Disorders Identification Test [25], and a total score was summed by 10 items including the frequency and amount of alcohol use and symptoms of dependence. Scores of ≥20 indicated harmful and hazardous levels of alcohol use in need of treatment [26]. Obstructive sleep apnea (OSA) was screened by using the STOP-Bang questionnaire, and participants were defined as OSA if they scored ≥3 [27].

### Statistical analysis

All statistical analyses were performed using SPSS version 16.0 (Chicago, SPSS Inc.). Differences in continuous variables were tested using *F* tests or *t* tests and differences in categorical variables were assessed using the Pearson chi-square test. The DSQL score representing quality of life was the dependent variable. The associations between sleep quality, anxiety symptoms, and quality of life were determined using binary logistic regression. The correlations between PSQI and DSQL scores, and between SAS and DSQL scores were used Pearson Correlation. The results were stratified in terms of sleep quality (good sleep quality = a PSQI global score < 7, equal to 0, versus poor sleep quality = a PSQI global score ≥ 7, equal to 1) and anxiety (anxiety symptoms = an GAD-7 score ≥ 10 versus few symptoms = GAD-7 < 10), and were adjusted for age (continuous), sex (males or females), educational level (less than high school, high school, or greater), marital status, physical activities (yes or no), net household income (continuous), cigarette smoking (yes or no), alcohol consumption (yes or no), years since diabetes diagnosis (continuous), comorbidities (yes or no), diabetic complications (yes or no), insulin use (yes or no), glycemic control (yes or no), and DSQL score (A DSQL score < 40 was represented as equal to 0 and a DSQL score ≥ 40 was represented as equal to 1). A cross-product interaction term was included in the logistic regression model to assess multiplicative interactions. Odds ratios (ORs) and 95% confidence intervals (CIs) were calculated using the contrast statement in SPSS 16.0. Variance was calculated using the Taylor series linearization method, which leads to an asymptotically unbiased estimate. All associations reported were statistically significant (*P* < 0.05) using two-tailed tests.

To estimate the biological interaction between sleep quality and anxiety, we created three new variables: 1) PSQI global score < 7 = no and anxiety = yes, versus others; 2) PSQI global score ≥ 7 = yes and anxiety = no, versus others; and 3) PSQI global score ≥ 7 = yes and anxiety = yes, versus others [28].

The relative excess risk due to interaction (RERI), the attributable proportion due to interaction (AP), and the synergy index (S) were used to estimate biological interactions [28]. The RERI is the excess risk attributed to interaction relative to the risk without exposure. AP refers to the attributable proportion of disease caused by interaction in subjects with both exposures. S is the excess risk from both exposures when there is a biological interaction relative to the risk from both exposures without interaction. In the absence of additive interactions, RERI and AP are equal to 0 [28]. The current study refined the criteria as a statistically significant RERI > 0, AP > 0, or S > 1 to indicate biological interactions.

## Results

### General participant characteristics

The mean age of participants was 64.1 ± 10.2 years, and 61.3% were female. The mean age of non-respondents was 63.8 ± 10.1 years, and 59.9% were female. There were no significant differences between participants and non-respondents (*P* > 0.05). The average DSQL score was 50.7 ± 12.9, and males had lower DSQL scores than females (48.8 ± 12.7 vs. 51.9 ± 13.1, *P* < 0.01). A total of 19.9% of the sample reported having good quality of life (a DSQL score < 40). A total of 17.3% participants were smokers, 11.9% were alcohol drinkers, 64.2% did not have any comorbidity, and 12.4% were treated with insulin. Participants had an average duration of 5.6 ± 5.1 years since Type 2 diabetes mellitus diagnosis. The distribution of participants’ general characteristics is presented in Table 1.

**Table 1:** Distribution of participants’ general characteristics according to quality of life

Variables	Good *n* = 198	Poor *n* = 799	*P*
Age > 60	61.7 ± 8.7	65.5 ± 10.9	< 0.001
Above high school	20 (10.1)	67 (8.4)	0.555
No spouse	27 (13.6)	131 (16.4)	0.284
Regular exercise	174 (87.9)	622 (77.8)	0.003
Income below population average (%)	100 (50.5)	416 (52.1)	0.799
BMI, mean (SD)	24.1 ± 2.5	23.8 ± 2.9	0.193
Median of disease duration (years)	3.8 ± 3.6	6.1 ± 5.3	< 0.001
Comorbidities	49 (24.7)	308 (38.5)	0.001
Complications	14 (7.1)	75 (9.4)	0.286
Smokers	39 (19.7)	133 (16.6)	0.328
Drinkers	36 (18.2)	83 (10.4)	0.004
Using insulin	7 (3.5)	117 (14.6)	< 0.001
Depression	52 (26.3)	348 (43.6)	< 0.001
Anxiety	90 (45.5)	562 (70.3)	< 0.001
PSQI> 7	33 (16.7)	302 (37.8)	< 0.001
HbA1c < 7%	63 (31.8)	110 (13.8)	< 0.001

*BMI* Body mass index, *HbA1c* Glycated hemoglobin, *DSQL* Diabetes Specificity Quality of Life Scale. Comorbidity refers to disease accompanying diabetes; complication refers to a disease caused by diabetes, *PSQI* Pittsburgh Sleep Quality Index

### Rates of poor sleep quality and anxiety symptoms

The prevalence of poor sleep quality was 33.6%. The rate of poor sleep quality was 16.7% (33/198) in participants with good quality of life and 37.8% (302/799) in participants with poor quality of life (χ^2^ = 30.74, *P* < 0.01). Among the participants, 65.4% had anxiety symptoms. The prevalence of anxiety symptoms was 45.5% (90/198) in participants with good quality of life and 70.3% (562/799) in those with poor quality of life (χ^2^ = 40.39, *P* < 0.01). The rates of poor sleep quality and anxiety symptoms were lower in participants with good quality of life than in those with poor quality of life (Table 1).

The correlations between PSQI and DSQL scores, and between GAD-7 and DSQL scores were positive, with correlation coefficients of 0.415 and 0.386, respectively (all *P* < 0.001).

### Biological interaction of sleep quality and anxiety symptoms on quality of life

The results from the multiple logistic regression models indicate the interaction using a combined effects method, with the *P* value of the interaction term indicating statistical significance of multiplicative interactions (Table 2). Participants with both poor sleep quality and anxiety symptoms had a significantly increased risk of reduced health status as measured by DSQL scores compared with those with neither poor sleep quality nor anxiety (OR: 7.45; 95% CI: 4.54–11.02; *P* < 0.001). In addition, those with poor sleep quality with no anxiety symptoms had a significantly higher risk of reduced health status as indicated by DSQL scores compared with those with neither poor sleep quality nor anxiety (OR: 3.48; 95% CI: 2.22–7.31; *P* < 0.001). Anxious participants who were good sleepers also had reduced health status compared with those who had neither poor sleep quality nor anxiety (OR: 2.55; 95% CI: 1.76–4.06; *P* < 0.001; (Table 2).

**Table 2:** Odds ratios for the association between sleep quality and quality of life by anxiety symptoms in participants with Type 2 DM

Sleep quality	Anxiety symptoms	Quality of life		
		Good (*n* = 198)	Poor (799)	OR^a^ (95% CI)
Good	< 50	91	147	1
Good	≥50	74	350	2.55 (1.76–4.06)
Poor	< 50	16	90	3.48 (2.22–7.31)
Poor	≥50	17	212	7.45 (4.54–11.02)

^a^Models were adjusted for age, sex, education level, marital status, exercise, income, BMI, disease duration, comorbidity, complications, smoking, drinking, insulin use, and depression

There was an additive interaction between poor sleep quality and anxiety symptoms (RERI, 2.27; 95% CI: 1.09–4.87; Table 3). Therefore, the OR of reduced DSQL score in participants with Type 2 diabetes mellitus and anxiety symptoms is 2.27 times higher as a result of the additive interaction compared with participants with good sleep quality and no anxiety, with 29% of declining health status attributed to the interaction between poor sleep quality and anxiety.

**Table 3:** Estimates of biological interaction between sleep quality and anxiety symptoms and its effects on quality of life in participants with Type 2 DM

Measures of biological interaction	Estimate (95% CI)
RERI	2.27 (1.09–4.87)
AP	0.29 (0.12–0.43)
S	1.46 (1.22–1.96)

Reference group is PSQI score > 7 and anxiety symptom score < 50

Models were adjusted for age, sex, education level, marital status, exercise, income, BMI, disease duration, comorbidity, complications, smoking, drinking, insulin use, and depression

## Discussion

We found that poor sleep quality was associated with a reduced DSQL score in participants with Type 2 diabetes mellitus. The addition of anxiety symptoms further augmented the decreased outcomes in participants with poor sleep quality, suggesting a greater reduction in quality of life in participants with both poor sleep quality and anxiety than in participants experiencing only poor sleep quality or only anxiety. To the best of our knowledge, this is the first study to elucidate the effect of poor sleep quality plus anxiety on these variables.

Several possible mechanisms may explain the combined effects of poor sleep quality and anxiety on quality of life reduction in participants with Type 2 diabetes mellitus. First, previous work suggests a positive significant association of PSQI global scores with anxiety [29]. Both factors affect quality of life of people with diabetes through biological pathways. Poor sleep quality and anxiety both activate the hypothalamic-pituitary-adrenal axis, stimulating sympathetic nervous system-adrenal responses, increasing platelet aggregation and inflammation, decreasing insulin sensitivity, and worsening glycemic control [30]. Poor sleep quality stimulates the central nervous system to secrete large amounts of catecholamines, which are released into the blood and lead to elevated blood sugar [31]. Research shows that poor glycemic control is related to reduced quality of life of people with diabetes [32].

Second, poor sleep quality and anxiety are both directly related to poor glycemic control [9, 33], poor sleep quality, decreased diabetes self-management, increased food intake, alterations in the timing and amount of food intake, preference for high energy food, impaired glucose tolerance and insulin sensitivity [34, 35]. Conversely, a chronic high fat, high protein diet and delicious food can increase fasting blood glucose, leading to anxiety [36] Anxiety disorders correlate directly with poor adherence to treatment, inadequate glycemic control, and increased adrenergic activity [9] In addition, research indicates that low fat diets can improve the quality of life of people with Type 2 diabetes mellitus [37].

Anxiety and poor sleep quality have a combined effect on glycemic control, which decreases the quality of life of people with Type 2 diabetes mellitus. These findings suggest that a combination of poor sleep quality and anxiety may reduce the quality of life of people with Type 2 diabetes mellitus.

Our results have substantial significance for public health. In the fully adjusted models, we estimated that 29% of the reduced quality of life of participants with Type 2 diabetes mellitus can be explained by an interaction between poor sleep quality and anxiety symptoms. The findings suggest that clinicians treating anxiety complaints in patients with Type 2 diabetes mellitus should screen for poor sleep quality, and vice versa. Efficacious behavioral and pharmacologic treatments exist for both anxiety disorders and poor sleep quality in people with Type 2 diabetes mellitus [38, 39]. Therefore, it is necessary to identify and implement best practice into routine health care in order to integrate health services for comorbid anxiety and diabetes in different types of service and in different countries. The application of either an anxiety- or sleep-focused intervention could produce relief for both symptoms, maximizing improvements in people’s quality of life. Given the difficulty in treating poor sleep quality or anxiety in certain people, recommendations to prevent these health problems are an inexpensive and practical means of improving quality of life in people with Type 2 diabetes mellitus.

Limitations of this study include the reliance on self-reported measures of poor sleep quality and anxiety levels. In addition, the cross-sectional nature of this sample means that the temporal order of causality cannot be inferred. Future research needs to address these limitations. Third, although we used reliable and valid measures of sleep quality and anxiety, these measures did not constitute clinical diagnoses of poor sleep quality and anxiety disorder. Future research should use clinical measures of sleep quality and anxiety to examine their effects on quality of life. Fourth, other confounding factors, such as hypoglycemia, dietary lifestyle, could not be adjusted in this study. Future research should consider the effects of adjusted dietary lifestyle on quality of life, and the effectiveness of sleeping and psychological intervention. Fifth, our sample was limited to Chinese participants; our findings need to be replicated in other ethnic populations.

## Conclusions

Poor sleep quality and anxiety symptoms all reduce quality of life in participants with Type 2 diabetes mellitus. Moreover, there was a combined effect of poor sleep quality and anxiety on reduced quality of life. As poor sleep quality and anxiety are common symptoms, the appropriate identification and treatment of these symptoms in people with Type 2 diabetes mellitus is very important to improve their quality of life.

## Data Availability

Please contact author for data requests.

## Abbreviations

T2DM: Type 2 diabetes mellitus
PSQI: Pittsburgh Sleep Quality Index
PHQ-9: Patient Health Questionnaire-9
GAD-7: General Anxiety Disorder questionnaire
DSQL: The Diabetes Specificity Quality of Life Scale
HbA1c: Glycated hemoglobin
BMI: Body mass index
OSA: Obstructive sleep apnea
Ors: Odds ratios
CIs: Confidence intervals
RERI: Relative excess risk due to interaction
AP: Attributable proportion due to interaction
S: Synergy index
